# Do low-income neighbourhoods have the least green space? A cross-sectional study of Australia’s most populous cities

**DOI:** 10.1186/1471-2458-14-292

**Published:** 2014-03-31

**Authors:** Thomas Astell-Burt, Xiaoqi Feng, Suzanne Mavoa, Hannah M Badland, Billie Giles-Corti

**Affiliations:** 1 School of Science and Health, University of Western Sydney, Sydney, Australia; 2 School of Geography and Geosciences, University of St Andrews, St Andrews, UK; 3 Centre for Health Research, School of Medicine, University of Western Sydney, Sydney, Australia; 4 The McCaughey VicHealth Centre for Community Wellbeing, School of Population and Global Health, University of Melbourne, Melbourne, Australia

**Keywords:** Australia, Environment, Green space, Health, Income, Inequity, Neighbourhood, Public open space

## Abstract

**Background:**

An inequitable distribution of parks and other ‘green spaces’ could exacerbate health inequalities if people on lower incomes, who are already at greater risk of preventable diseases, have poorer access.

**Methods:**

The availability of green space within 1 kilometre of a Statistical Area 1 (SA1) was linked to data from the 2011 Australian census for Sydney (n = 4.6 M residents); Melbourne (n = 4.2 M); Brisbane (n = 2.2 M); Perth (n = 1.8 M); and Adelaide (n = 1.3 M). Socioeconomic circumstances were measured via the percentage population of each SA1 living on < $21,000 per annum. Negative binomial and logit regression models were used to investigate association between the availability of green space in relation to neighbourhood socioeconomic circumstances, adjusting for city and population density.

**Results:**

Green space availability was substantively lower in SA1s with a higher percentage of low income residents (e.g. an incidence rate ratio of 0.82 (95% confidence interval (95% CI) 0.75, 0.89) was observed for SA1s containing ≥20% versus 0-1% low income residents). This association varied between cities (*p* < 0.001). Adelaide reported the least equitable distribution of green space, with approximately 20% greenery in the most affluent areas versus 12% availability in the least affluent. Although Melbourne had a smaller proportion of SA1s in the top quintile of green space availability (13.8%), the distribution of greenery was the most equitable of all the cities, with only a 0.5% difference in the availability of green space between SA1s containing 0-1% low income households versus those with ≥20%. Inequity of access, however, was reported across all cities when using logit regression to examine the availability of at least 20% (odds ratio 0.74, 95% CI 0.59, 0.93) or 40% (0.45, 0.29, 0.69) green space availability in the more disadvantaged versus affluent neighbourhoods.

**Conclusion:**

Affirmative action on green space planning is required to redress the socioeconomic inequity of access to this important public health resource.

## Background

Characterised long ago as the “lungs of the city” [1], parks and other forms of green space are rapidly entering the policymakers tool kit as a lever for potentially enhancing health and narrowing health disparities [2-4]. Although findings are not unequivocal and evidence is largely based upon cross-sectional analyses of observational (i.e. non-experimental) data, a rapidly growing literature reports a variety of health benefits could result from exposure to green space [5-7]. For example, mounting evidence suggests that being within physical proximity, or even merely having visuals of green space can support mental health and promote restoration from stressful circumstances [8-11]. Some recent studies suggest that these effects on mental health may be closely entwined with active lifestyles [12-17] and have different influences between women and men [18]. A number of studies report people to greater levels of engagement in physical activity among residents of greener neighbourhoods, some of whom also benefitting from low body mass index [5,19-21], though these findings tend to vary by population sub-group [22], geographic contexts [23-25], and for particular types of physical activity [26]. It is often suggested in the literature that green spaces may promote social cohesion through providing places for people to meet [27,28], and, more recently, the possibility that a greener local environment can also assist in people getting healthier durations of sleep [29] If even just some of these reported benefits are apparent, promoting the availability and use of green space can be part of multi-sectoral initiatives aiming to reduce the burden of chronic diseases[30,31], promote longer, healthier lives and to narrow the health gap between rich and poor [32-34]. The availability of green space is, therefore, a potentially important preventive health resource [35] and public access to them needs to be protected [17].

This is good news for people who live near parks, but less helpful for those in communities with poor access to green space. Inequality is unlikely to come about by random chance since neighbourhoods containing greenery are often highly desirable [36-38] and more costly to buy into [39,40]. People on low incomes already shoulder the vast burden of preventable lifestyle-related health conditions. They have the most to gain from green spaces, yet may have the poorest access through a lack of purchasing power.

Do low income neighbourhoods have less green space? Some research has reported corroborative evidence that disadvantaged neighbourhoods lack proximity to green space [33,41], though other work has reported this social injustice is not universal across national contexts [42-44]. Nor is such an association necessarily consistent between cities within the same country. In Australia, a National Urban Policy was implemented in 2011 with the aim to nurture a ‘productive, sustainable and liveable future’ [3]. Yet, variations in built environment planning policies at the state-level may have resulted in differences in the equity of green space availability between cities that are difficult to predict. In Melbourne, for example, the ‘Victorian Planning Provisions’ (VPP) state local parks are to be located within a 400 m safe walking distance of at least 95% of all dwellings [45]. In Perth, by contrast, it is a requirement that 10% of all sub-divisible land is allocated to parks and other open spaces [46].

In Australia, a lack of green space data with nationwide coverage and harmonious definition has hitherto inhibited multi-city investigations of this important public health question. With data fitting this description now available, the purpose of this paper was to investigate for the first time to what extent green space availability is associated with neighbourhood socioeconomic circumstances within Australia’s biggest cities. We were particularly interested in specific minimum amounts of green space available across neighbourhood disadvantage and the variation between cities. This focus upon amount was due to and because of emerging evidence suggesting that while a little green space is beneficial for wellbeing, large amounts are more likely to promote healthy and active lifestyles [20,21].

## Method

### Setting

The study was set across Australia’s five most populous cities: Sydney (n = 4.6 M residents); Melbourne (n = 4.2 M residents); Brisbane (n = 2.2 M residents); Perth (n = 1.8 M residents); and Adelaide (n = 1.3 M residents) [47]. The five cities comprised 62% of the Australian population in 2011. City definitions were based upon the Australian Bureau of Statistics (ABS) ‘Urban Centres and Localities’ (UCL), which are part of the 2011 Australian Statistical Geography Standard (ASGS). Full details on the derivation of UCLs can be found elsewhere [48]. In brief, UCLs are aggregations of Statistical Areas Level 1 (SA1); the smallest geography at which the 2011 Australian Census was disseminated (~400 residents per SA1).

### Unit of analysis

SA1s were the primary unit of analysis in this study and are nested within Statistical Local Areas Level 2 (SA2). SA2s have a population of approximately 10,000 residents and are designed to geographically represent contiguous communities which interact together in social, economic and political terms [49]. Approximately 31 SA1s contribute to each SA2 within these cities. The provision of green space within an SA1 may be influenced by what is available within the larger SA2 (e.g. planning and access to local services), therefore the hierarchical clustering of SA1s within SA2s was accounted for within the analytical design (see *Statistical analysis).*

### Outcome variable: green space availability

Data on green space were extracted from the Australian Bureau of Statistics (ABS) 2011 Meshblocks [50] using Geographic Information Systems (GIS) [51]. The Meshblock is the smallest geographic unit in the ASGS and is the base unit for all the larger geographies, including SA1s (which comprise 6–7 meshblocks on average). Each Meshblock was classified by the ABS according to the dominant land-use: i) water; ii) parkland; iii) residential; iv) industrial; v) commercial; vi) education; vii) hospital/medical; viii) agricultural; ix) transport; and x) other. Meshblocks identified as ‘parkland’ formed the raw data of the outcome variable and had a mean area of 0.089 km^2^ (0.57 km^2^ standard deviation). Meshblocks identified as ‘agricultural’ were not considered within the outcome variable since those areas were not routinely publically accessible for recreation and physical activity. Domestic gardens are also not included in the ‘parkland’ category. To gain a general understanding of the spatial patterning of green space across each city, catchment areas of 1 kilometre radius were overlaid on the population-weighted centroid of each SA1. This allowed for the estimation of green space area (m^2^) and calculation as a percentage of the overall land-use available within a reasonable walking distance that was not inhibited by administrative boundaries. A catchment area approach defining green space availability has been demonstrably associated with health outcomes and active lifestyles by studies in Australia [21,22] and the Netherlands [52]. Previous work has found that the use of green space is particularly sensitive to distance [53]; hence a restriction of the catchment areas to a 1 km radius.

### Neighbourhood socioeconomic circumstances

Income data from the 2011 Australian Census were extracted for SA1s to calculate the percentage of an SA1 population living on a low income. The definition of low income was < $21,000 per annum, in line with that used in the SEIFA (Socio-Economic Index For Areas) composite indices of socioeconomic disadvantage [54]. We selected to focus on this income-based measure due to the simplicity of interpretation. This measure was initially modelled as a continuous variable. To investigate for potential curvilinear associations with green space, the percentage low income variable was classified into the following categories: 0%; 1-4%; 5-9%; 10-19%; ≥ 20% of low income residents per SA1.

### Other explanatory variables

Differences in the potential association between green space availability and neighbourhood socioeconomic circumstances could manifest between cities due to historical variation in regional urban planning policy. To investigate such a possibility, each city was controlled as a categorical variable. It was also plausible that any potential association between percentage green space and low income neighbourhoods could be confounded by population density, with space for parks within densely built environments at a premium. Although the focus of the study was on the five most populous cities and all SA1s were, by definition, of urban character, there remained substantive geographical heterogeneity in residential population density between SA1s within Central Business Districts (CBDs), along the coastline and throughout more distant suburbs. To control for this potential confounder, population counts were extracted from the 2011 Census and divided by the area (km^2^) of each SA1 to give a measure of population density. For modelling purposes, this variable was calculated as a natural logarithm as the data were skewed.

### Statistical analysis

A GIS map was generated to gain a visual understanding of the spatial patterning of green space across each city. Categories of percentage green space were selected for visualisation based upon historic planning policy in Western Australia, where it is a requirement that 10% of all sub-divisible land is allocated to parks and other open spaces [46]. As such, we chose to map green space across all five cities according to the following categories: (i) 0%; (ii) 1% to 9%; (iii) 10% to 19%; (iv) 20% to 39%; and (v) ≥40%. Cross-tabulations were used to describe and graph the distribution of these green space categories with respect to neighbourhood socioeconomic circumstance for each city.

The first step in the modelling strategy was to analyse the patterning of green space by neighbourhood socioeconomic circumstances. An Ordinary Least Squares regression model was ruled out for this purpose, as the percentage green space variable was highly skewed (i.e. not ‘normally distributed’). A Poisson model was investigated as an alternative. Poisson regression assesses count variables [55] and has been widely used to investigate the geographical patterning of mortality [56] and morbidity [57]. To operationalize the Poisson regression, the geographical area (m2, expressed as an integer with fully decimals rounded up) of green space was expressed as an integer and fitted as the dependent variable. The natural logarithm of the area of each 1 km buffer was fitted as an offset. A goodness of fit statistic calculated from an empty model indicated that the area of green space variable was significantly different from the Poisson distribution (chi2: 7.43e + 09, prob > chi2: < 0.0001). Descriptive diagnostics further supported this case, with the variance of the green space area variable (1.37e + 11 m2) substantively greater than the mean (455,061 m2). Negative binomial regression, used in previous studies of green space and health [32,33], was used as a substitute to the Poisson distribution to account for this over-dispersion. Robust standard errors [58] were used to adjust for the hierarchical data structure (n = 28,626 SA1s within n = 937 SA2s). Model parameters were exponentiated to incidence rate ratios (IRRs) and 95% confidence intervals (95% CI), wherein an IRR above 1 indicates a positive association and below 1 a negative association between the dependent and independent variables.

We fitted associations between percentage green space and each of the explanatory variables separately. Models were then built up, firstly with city as a categorical variable. This model was then augmented by population density, to adjust for between city differences in green space that could be explained by residential structure. The categorical measure of neighbourhood socioeconomic circumstance was then added to this model to explore whether association with green space could be identified independently to city and population density. Finally, to investigate whether any potential association between green space and neighbourhood circumstances varied from one city to another, the last stage of our analysis was to fit an interaction term between the socioeconomic and city variables.

The second step in the modelling strategy was to investigate different thresholds of the amount of green space availability within a 1 km Euclidean buffer using pre-defined binary variables. The purpose of this analysis was to account for different levels of green space access which may be critical for promoting health and active lifestyles [20,21]. To operationalize this investigation of thresholds, we constructed a suite of binary variables in line with the categories used in the mapping of green space in each city, denoting whether (or not) the population of an SA1 had access to at least 10%, 20% or 40% green space. Logit regression with robust standard errors and the same model building strategy was used to fit associations between each binary measure of green space access with the city variable, population density and neighbourhood socioeconomic circumstances. Logit regression parameters were exponentiated to odds ratios (ORs) and 95% CIs, wherein an OR and 95% confidence interval above 1 indicates a greater likelihood of a positive association and below 1 a greater likelihood of a negative association compared with the reference group.

The log-likelihood ratio test was used to identify statistically significant effects (p < 0.05). All analyses were conducted in 2013 using STATA IC/SE V.12 (StataCorp LP, College Station, TX, USA).

## Results

**Figure 1:**
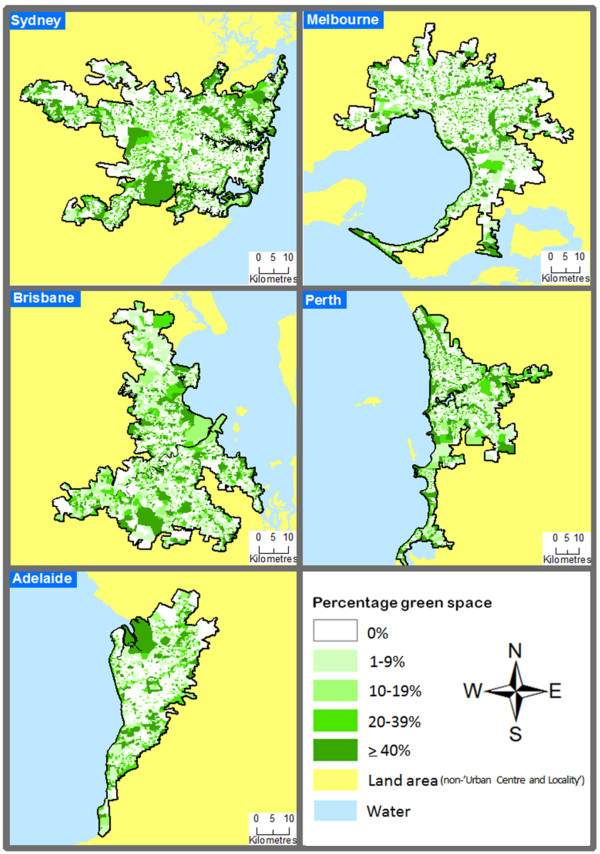
Spatial patterning of green space in Australia’s most populous cities.

Figure 1 illustrates the spatial patterning of green space was not uniform across each city. Evidence of green space clustering was visually apparent. Table 1 indicates Sydney and Melbourne accounted for the majority of SA1s in the dataset. While these cities were the most populous (as indicated in the Method section), Perth reported the highest mean of green space availability of all five cities (17.3%). Neighbourhoods containing approximately zero percentage green space were in the minority, while areas with ≥40% greenery were rarer in Melbourne compared to the other cities. Some variation between cities was also apparent in terms of the distribution of low income neighbourhoods. For example, 9.1% and 10.4% of neighbourhoods in Sydney and Brisbane had approximately 0% low income households, whereas Adelaide only had 3.2%. Adelaide also had the highest percentage of neighbourhoods in the ≥20% low income households category at 13.8%, in comparison to Perth and Brisbane with 4.2% and 5.2% respectively.

**Table 1:** Descriptive statistics: percentage green space and low income households, by city

	**Total**	**Sydney**	**Melbourne**	**Brisbane**	**Perth**	**Adelaide**
N SA1s (%)	28,626	9,286 (32.4%)	8,600 (30.0%)	4,448 (15.5%)	3,699 (12.9%)	2,593 (9.1%)
Mean percentage green space (standard deviation)	15.1% (12.2%)	16.8% (13.2%)	12.7% (10.7%)	12.7% (10.7%)	17.3% (12.8%)	13.3% (11.8%)
Percentage green space (categories)	N SA1s (%)					
0%	409 (1.4%)	52 (0.6%)	180 (2.1%)	57 (1.3%)	29 (0.8%)	91 (3.5%)
1% – 9%	11,596 (40.5%)	3,237 (34.9%)	4,307 (50.1%)	1,661 (37.3%)	1,208 (32.7%)	1,183 (45.6%)
10% – 19%	9,537 (33.3%)	3,307 (35.6%)	2,587 (30.1%)	1,591 (35.8%)	1,284 (34.7%)	768 (29.6%)
20% – 39%	5,710 (20.0%)	2,108 (22.7%)	1,254 (14.6%)	944 (21.2%)	956 (25.8%)	448 (17.3%)
≥ 40%	1,374 (4.8%)	582 (6.3%)	272 (3.2%)	195 (4.4%)	222 (6.0%)	103 (4.0%)
Mean percentage low income households^a^ (standard deviation)	9.1% (7.1%)	8.8% (7.5%)	9.5% (7.0%)	9.5% (7.0%)	8.4% (6.0%)	11.8% (8.0%)
Percentage low income households^a^ (categories)	N SA1s (%)					
0%	2,155 (7.5%)	844 (9.1%)	511 (5.9%)	462 (10.4%)	254 (6.9%)	84 (3.2%)
1 – 4%	6,109 (21.3%)	2,139 (23.0%)	1,548 (18.0%)	1,187 (26.7%)	873 (23.6%)	362 (14.0%)
5 – 9%	9,776 (34.2%)	3,126 (33.7%)	3,056 (35.5%)	1,500 (33.7%)	1,366 (36.9%)	728 (28.1%)
10 – 19%	8,621 (30.1%)	2,468 (26.6%)	2,976 (34.6%)	1,067 (24.0%)	1,049 (28.4%)	1,061 (40.9%)
20%+	1,965 (6.9%)	709 (7.6%)	509 (5.9%)	232 (5.2%)	157 (4.2%)	358 (13.8%)

^a^low income household is defined as having a household income < $21,000 in the 2011 Australian census.

Table 2 shows the results of the negative binomial regression modelling to assess the patterning of green space availability by neighbourhood socioeconomic circumstance, controlling for city and population density. Model 1 indicates the average area of green space between SA1s in Perth was not substantively different to Sydney, though lower mean areas of green space were reported in Melbourne, Brisbane and Adelaide. As expected, green space was rarer in neighbourhoods with a higher population density. Adding in the percentage low income measure (Model 2) revealed an independent negative association between the area of green space and neighbourhood socioeconomic circumstance. For example, an IRR of 0.82 (95% CI 0.75, 0.89) suggests that the neighbourhoods containing ≥20% low income residents contained 18% less green space in comparison to those with 0-1% low income residents (*p* < 0.001).

**Table 2:** **Association between green space area (m**^**2**^**) and neighbourhood socioeconomic circumstances, adjusting for city and population density: Negative binomial regression with robust standard errors, using total neighbourhood area (m**^**2**^**) as an offset**

	**Model 1**	**Model 2**
	**Incidence rate ratio (95% Confidence interval)**	
City (ref: Sydney)		
Melbourne	0.73 (0.67, 0.80)***	0.75 (0.68, 0.81)***
Brisbane	0.84 (0.77, 0.93)***	0.85 (0.77, 0.93)***
Perth	0.94 (0.86, 1.04)	0.96 (0.87, 1.05)
Adelaide	0.73 (0.63, 0.84)***	0.75 (0.66, 0.87)***
Population density (logged)	0.87 (0.84, 0.89)***	0.87 (0.85, 0.89)***
Percentage low income households^a^ (ref: 0%)		
1 – 4%		0.97 (0.92, 1.02)
5 – 9%		0.88 (0.84, 0.94)***
10 – 19%		0.80 (0.75, 0.85)***
20%+		0.82 (0.75, 0.89)***

^a^low income household is defined as having a household income < $21,000 in the 2011 Australian census.

*** *p* < 0.001; ** *p* < 0.01; * *p* < 0.05.

**Figure 2:**
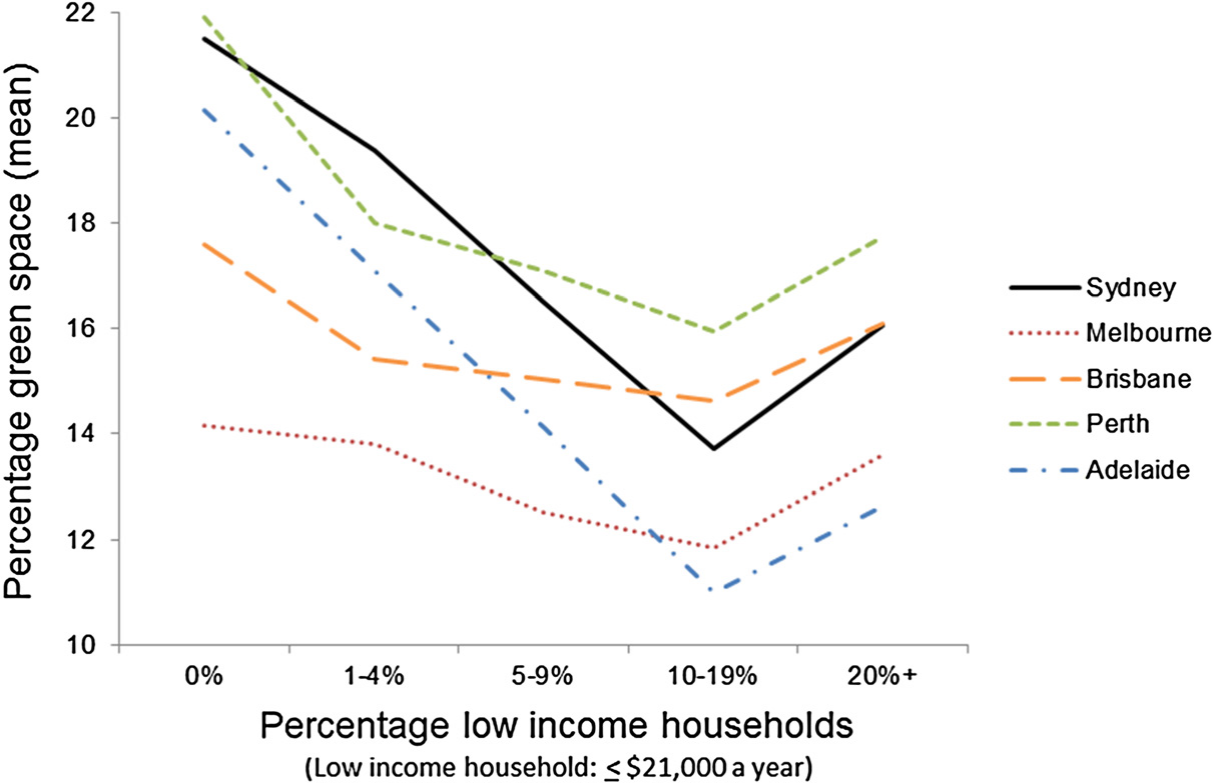
Patterning of green space by neighbourhood socioeconomic circumstance and city.

An interaction term suggested there were systematic differences in the level of association between green space availability and neighbourhood socioeconomic circumstance between each city (*p* = 0.0006 for the trend). Figure 2 shows the extent of this interaction, with a clear patterning of green space by neighbourhood socioeconomic circumstance across most cities in the sample. The steepest gradients were for Sydney and Adelaide, but a modest negative association for Melbourne was indicative of a relatively more equitable distribution of green space.

**Figure 3:**
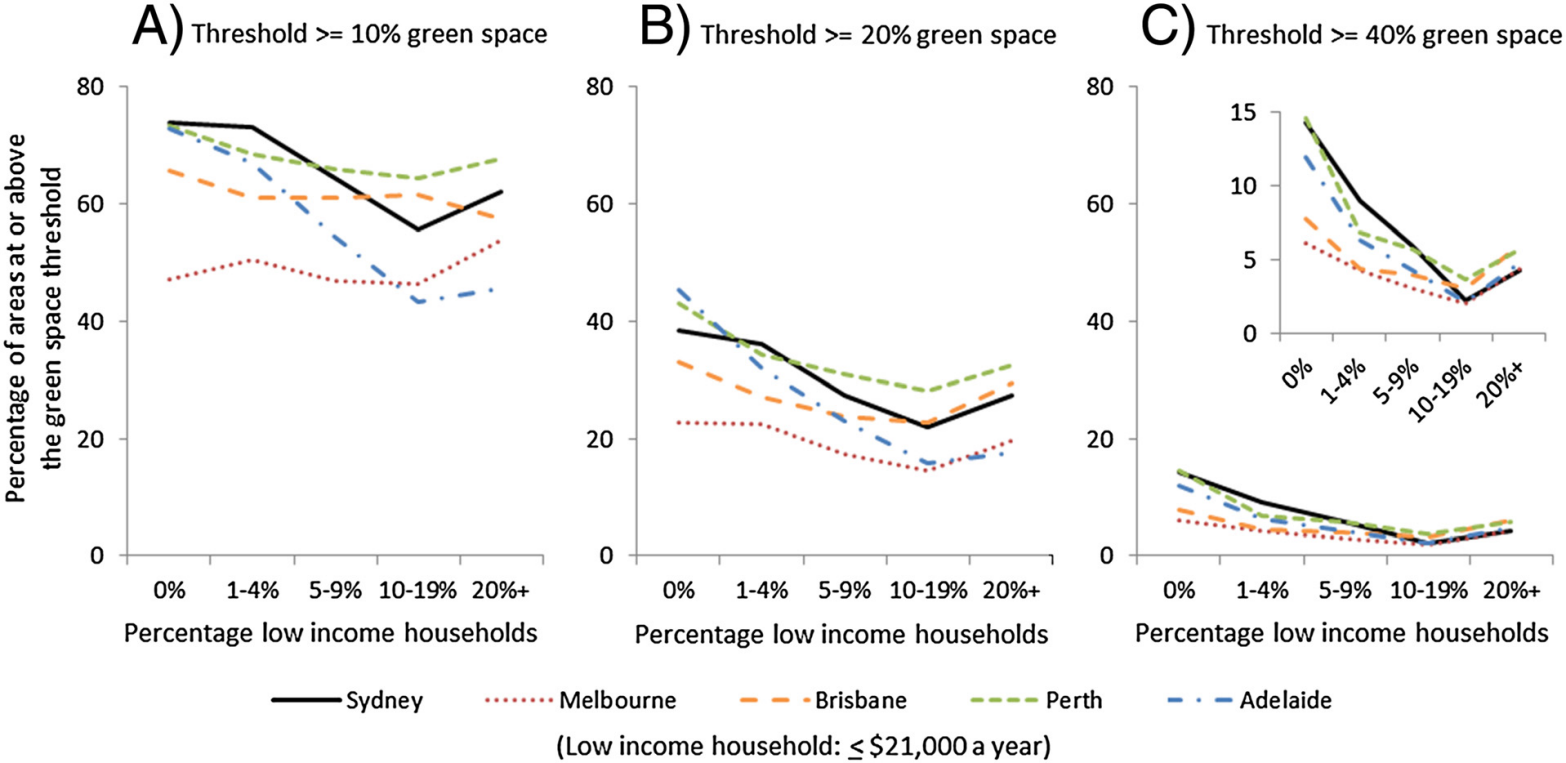
**Patterning of green space by neighbourhood socioeconomic circumstance and city, using three binary definitions of green space availability.** Panel **A**: Outcome >=10% green space. Panel **B**: Outcome >=20% green space. Panel **C**: Outcome >=40% green space (enlarged inset).

The final set of analyses made use of the binary definitions of availability for an explicit focus on green space thresholds. Figure 3 reports the contrasting patterns for each binary variable of green space amount by neighbourhood socioeconomic circumstances across each city. Graph A shows lower income neighbourhoods within Perth, Brisbane, Sydney and (especially) Adelaide were less likely to have at least 10% green space. The opposite trend was found in the city of Melbourne; lower income neighbourhoods were more likely to have at least 10% green space. A similar pattern was observed once the green space threshold was increased to at least 20% (Graph B) and at least 40% (Graph C). Lower income neighbourhoods with a minimum of 20% or 40% green space were less common across all cities.

Table 3 shows that the overall patterning of green space by neighbourhood socioeconomic circumstances was robust to controls for city and population density. The magnitude of this association, however, tended to be stronger as the minimum percentage threshold of green space was increased from ≥10% to ≥20% and especially at ≥40%. For the definition of at least 10% green space, a negative association was evident. The same interaction found in our earlier models was also observed, with the patterning of green space by low income neighbourhoods in Melbourne inconsistent with the overall negative trend (*p* < 0.0001).

**Table 3:** Association between minimum percentage green space thresholds and neighbourhood socioeconomic circumstances, adjusting for city and population density: binary logit regression with robust standard errors

	**Model 1**	**Model 2**	**Model 3**
	**Odds Ratio (95% Confidence Interval)**		
% green space cut-point for the outcome variable	≥10%	≥20%	≥40%
City (ref: Sydney)			
Melbourne	0.48 (0.39, 0.59)***	0.49 (0.39, 0.62)***	0.45 (0.30, 0.67)***
Brisbane	0.75 (0.60, 0.94)*	0.68 (0.53, 0.87)**	0.47 (0.29, 0.74)***
Perth	0.96 (0.75, 1.24)	0.95 (0.74, 1.22)	0.72 (0.47, 1.09)
Adelaide	0.53 (0.40, 0.72)***	0.61 (0.44, 0.84)**	0.58 (0.32, 1.06)
Population density (logged)	0.82 (0.77, 0.87)***	0.74 (0.70, 0.78)***	0.61 (0.57, 0.65)***
Percentage low income households^a^ (ref: 0%)			
1 – 4%	1.04 (0.92, 1.17)	1.00 (0.88, 1.13)	0.85 (0.69, 1.06)
5 – 9%	0.85 (0.74, 0.97)*	0.76 (0.66, 0.88)***	0.64 (0.50, 0.81)***
10 – 19%	0.73 (0.62, 0.85)***	0.61 (0.51, 0.73)***	0.35 (0.26, 0.47)***
20%+	0.77 (0.63, 0.93)**	0.63 (0.51, 0.79)***	0.30 (0.20, 0.44)***

^a^low income household is defined as having a household income < $21,000 in the 2011 Australian census.

***p < 0.001; **p < 0.01; *p < 0.05.

Increasing the threshold of availability to at least 20% green space, negative gradients with neighbourhood socioeconomic circumstances were observed for all cities including Melbourne. The magnitude of association between green space availability and neighbourhood socioeconomic circumstances was amplified when a ≥40% green space threshold was considered. This definition prioritised areas with a substantial amount of green space, and no evidence of variation in the association with neighbourhood socioeconomic circumstances was evident across cities.

## Discussion

Previous work has suggested that built environment health promoting resources like green spaces are, within some contexts, inequitably distributed with respect to populations at high risk of lifestyle-related chronic diseases [33,41-43], though not all [44]. Results from our study indicate, for the first time, a similar relationship between green space availability and neighbourhood socioeconomic circumstance exists within Australia’s most populous cities. Neighbourhoods in Sydney, Melbourne, Brisbane, Perth and Adelaide with a higher percentage of low income households had substantively less green space availability. What this means is that Australians who are most at risk of preventable chronic health issues, like obesity, cardiovascular disease and type 2 diabetes mellitus, live in environments that contain the least green space for supporting positive lifestyle modification. Indeed, the geographical clustering of less favourable socioeconomic circumstances and poor green space availability could constrain the effectiveness of multiple lifestyle interventions in high risk populations that are otherwise proven to work [59]. Although it could be argued that living within close proximity to parks and other greenery does not necessarily guarantee use (e.g. for physical activity), the results of our study suggest that opportunities to make use green spaces are fewer among the residents of low income neighbourhoods. Thus, to give public health interventions the best possible chance of success, a recommendation for planners, policymakers and local communities is to devise mechanisms for affirmative action that address the socioeconomic inequity of green space availability by making more accessible specifically in low income neighbourhoods.

A second finding from our study was that the magnitude of association between green space and neighbourhood socioeconomic circumstances varied between cities. The gradient was steepest in the cities of Sydney and Adelaide, but relatively flatter in Melbourne. As the distribution of low-income neighbourhoods varies between cities, these results suggest that investments to equalise green space availability should be city-specific. Moreover, it was notable that for some cities (e.g. Adelaide and Sydney), average levels of green space availability were slightly higher in the ≥ 20% low income households category in comparison to those SA1s containing 10-19% low income households. This may be related to urban sprawl and suburbs which are more distant from central business districts having cheaper land and more green space. Further investigation of how green space equity varies between and within cities in relation to urban sprawl and related phenomena (e.g. transport infrastructure) is warranted [60].

These recommendations are supported by the third major result of the study. We noted a lower proportion of low income neighbourhoods in Sydney, Brisbane, Perth and Adelaide had at least 10% green space availability. Intriguingly, the opposite trend was found in Melbourne, where a greater proportion of low income neighbourhoods had at least 10% green space availability. This Melbourne-specific result could be construed as a rare occurrence wherein more disadvantaged groups have better access to a health promoting resource. We do not endorse this conclusion for two reasons. First, due to an absence of data, our study does not account for differences in the type and quality of green space, such as the difference between a public park and a private golf course. Some green spaces may be more health promoting than others due to the range of opportunities and infrastructure they afford to different population groups (e.g. playgrounds for young children, well maintained footpaths of sufficient width to allow pushchair and wheelchair access, more aesthetically pleasing, reasonably level topography that does not unduly increase the risk of falls in older adults) [61,62]. Previous work has suggested that green spaces within disadvantaged neighbourhoods are often of poorer quality than those in more affluent areas [63,64]. This requires further investigation, as higher quality green space is associated with increased recreational walking [65].

Second, if a minimum of 10% green space availability is used, there is a danger that low income neighbourhoods in Melbourne will be wrongly ignored for investments in green space planning policy. Our study demonstrates that larger amounts of green space, whether a minimum of 20% or 40% land-use, were rarer in low income neighbourhoods across all the cities (including Melbourne). Larger amounts of green space are more supportive of active lifestyles [20,21] and, therefore, may be more important for promoting healthier communities. However, it is important to re-emphasise that the focus of this study was on the provision of green space across neighbourhoods of different socioeconomic circumstances. It cannot be deduced from this study that making more green space available in low income neighbourhoods will result in uses of those amenities in a way that promotes health, nor is it possible to make predictions about whether levels of use will vary by socioeconomic circumstances; longitudinal studies tracking change in neighbourhood environments among people who remain in-situ are needed to answer these questions. Previous epidemiological research has reported higher rates of poor health in low income neighbourhoods containing more green space [66] while qualitative work notes that intentions to use green space are determined only in part by its availability [67]. Thus, in calling for affirmative action to equalise the availability of green space, it is important to consider this as only the first step in a built environment strategy for preventive health that will also need to engage with local communities to understand motivations and to promote use of existing green spaces.

Strengths of our study include a consistent objective definition of green space available across the five most populous cities in Australia. These data open up the possibility of further nationwide research on green space and health, which has been previously focussed upon specific regions or cities. The modelling of variables for small geographical areas afforded the detection of subtleties in the association between green space and socioeconomic circumstances that exist within and between cities. This is a strength as it is the locally available green spaces that are most likely to play a role in community health promotion [53]. This focus upon the ‘local’ does not permit inferences on to what extent the overall level of green space for larger areas (e.g. cities) may be related to health; indeed, a recent paper which reports higher rates of all-cause mortality in greener US cities [60] demonstrates the importance of differentiating between studies according to the geographical scale of the analytical unit.

Another strength was the green space data were temporally consistent with demographic population data obtained from the most recent Australian Census (2011). A limitation of this green space measure was that it was not sensitive to differences in quality, nor did it detect solitary trees and green canopy within highly urbanised areas unless they were within a park. It is therefore possible that our study underestimates the prevailing level of socioeconomic inequity in green space availability. Conversely, if private types of green space (e.g. golf courses) are more plentiful in affluent surroundings, it is possible that the socioeconomic inequity in publically-accessible green space within our study is less severe than reported. An important next step for the development of a nationwide measure of green space will be to push beyond quantity and to distinguish between different types and features.

Lastly, the conventional method of assessing neighbourhood socioeconomic circumstances in Australia is by using the SEIFA indices [54], which take into account income, education, employment, occupation and housing circumstances. Relevant measures for 2011 were not in circulation at the time of our data analysis, but have since become available. We conducted sensitivity analyses with the Index of Relative Socioeconomic Advantage and Disadvantage (IRSAD), which takes into account the mix of affluence and deprivation present within an SA1, which revealed very similar results to those reported in our study.

## Conclusions

Major chronic health problems such as obesity and type 2 diabetes mellitus are preventable but pose a daunting future for healthcare systems internationally [68,69]. Our study shows the low income neighbourhoods that shoulder much of the burden of these preventable diseases have less green space. While public access to existing green spaces should be promoted and protected, the key message for planners and policymakers from this study is that affirmative action is required with large-scale investments in green space initiatives targeting low income neighbourhoods if we are to build healthy environments for all.

## Competing interests

The authors declare that they have no competing interests.

## Authors’ contributions

TAB initiated the project, contributed to the development and design of the study, conducted the statistical analyses and wrote the first draft of the manuscript. XF contributed to the development and design of the study, the statistical analyses and redrafting of the manuscript. SM, HB and BGC contributed to the development and design of the study, interpretation of the statistical analyses and redrafting of the manuscript. All authors read and approved the final version of the manuscript.

## Pre-publication history

The pre-publication history for this paper can be accessed here:

http://www.biomedcentral.com/1471-2458/14/292/prepub
